# Antimicrobial Resistance Mechanisms among *Campylobacter*

**DOI:** 10.1155/2013/340605

**Published:** 2013-06-24

**Authors:** Kinga Wieczorek, Jacek Osek

**Affiliations:** Department of Hygiene of Food of Animal Origin, National Veterinary Research Institute, Partyzantow 57, 24-100 Pulawy, Poland

## Abstract

*Campylobacter jejuni* and *Campylobacter coli* are recognized as the most common causative agents of bacterial gastroenteritis in the world. Humans most often become infected by ingesting contaminated food, especially undercooked chicken, but also other sources of bacteria have been described. Campylobacteriosis is normally a self-limiting disease. Antimicrobial treatment is needed only in patients with more severe disease and in those who are immunologically compromised. The most common antimicrobial agents used in the treatment of *Campylobacter* infections are macrolides, such as erythromycin, and fluoroquinolones, such as ciprofloxacin. Tetracyclines have been suggested as an alternative choice in the treatment of clinical campylobacteriosis but in practice are not often used. However, during the past few decades an increasing number of resistant *Campylobacter* isolates have developed resistance to fluoroquinolones and other antimicrobials such as macrolides, aminoglycosides, and beta-lactams. Trends in antimicrobial resistance have shown a clear correlation between use of antibiotics in the veterinary medicine and animal production and resistant isolates of *Campylobacter* in humans. In this review, the patterns of emerging resistance to the antimicrobial agents useful in treatment of the disease are presented and the mechanisms of resistance to these drugs in *Campylobacter* are discussed.

## 1. Introduction

*Campylobacter *microorganisms are small (0.2–0.9 *μ*m wide and 0.2–5.0 *μ*m long), spirally curved, and motile Gram-negative bacteria that are commonly present in the intestinal tract of domestic and wild animals [1]. Twenty-one *Campylobacter *species have been identified and characterized so far and among them, the most important pathogenic species being *Campylobacter jejuni *and, to a lesser extent, *Campylobacter coli*. Both these *Campylobacter* species are different from other pathogens associated with food-borne disease since they are essentially microaerophilic, able to grow in an atmosphere containing approximately 10% CO_2_ and 5% O_2_, at a narrow temperature range between ca. 30°C and 46°C, and thus classified as thermophilic campylobacters [2].

In humans, *Campylobacter* bacteria cause illness called campylobacteriosis, which is the most common human gastroenteric infection in developed countries [1]. *Campylobacter *is responsible for diarrhoea in an estimated 400–500 million people globally each year [2, 3]. Some of the *Campylobacter *species are zoonotic pathogens (mainly *C. coli*, *C. jejuni*) and humans most often become infected by ingesting contaminated food or water. The main sources of these microorganisms are raw or uncooked meat, especially poultry meat, unpasteurized milk, contaminated drinking water, and contact with animals [1, 2]. Human-to-human spread has also been observed, although at low frequencies [4]. The illnesses are usually sporadic, although outbreaks may occur. The infectious dose is not exactly determined, but disease has been experimentally induced with as few as 500 bacterial cells [5]. Furthermore, a clinical trial with a volunteer experimental infection model with a well-characterized outbreak *C. jejuni* CG8421 strain has been performed [6]. In this experiment, a total of 23 subjects received 1 × 10^6^ or 1 × 10^5^ colony forming units of *C. jejuni* with attack rates (percentage of patients who become ill) of 100% and 93%, respectively. The infective dose depends on a number of factors including the vehicle in which it is ingested and the susceptibility of the individuals. In children, the number of bacteria responsible for the illness may be lower than it is in adults [7, 8]. After an incubation period of 1–5 days, symptoms, including diarrhoea, abdominal pain, and fever, appear. Campylobacteriosis is normally a self-limiting disease, but in some cases complications may occur, such as reactive arthritis (in 1- to 5% of *Campylobacter *infected patients) and Guillain-Barré syndrome, a postinfectious polyneuropathy that is a leading cause of paralysis (in 0.01–0.03% of *Campylobacter *enteritis patients) [9–11]. 

In the management of human campylobacteriosis, fluid therapy is the most important. Antimicrobial treatment is needed only in patients with more severe disease and in those who are immunologically compromised. The most common antimicrobial agents used in the treatment of *Campylobacter *infections are macrolides, such as erythromycin, and fluoroquinolones, such as ciprofloxacin [1]. Tetracyclines have been suggested as an alternative choice in the treatment of clinical campylobacteriosis, but in practice they are not often used.

## 2. Antimicrobial Resistance Mechanisms in *Campylobacter*

Antimicrobial resistance in bacteria originated from food of animal origin, including *Campylobacter*, has become in recent years a major public health concern in both developed and developing countries [12, 13]. An increasing numbers of *Campylobacter* isolates have developed resistance to fluoroquinolones and other antimicrobials such as macrolides, aminoglycosides, and betalactams. Furthermore, intrinsic resistance in *C. jejuni *and *C. coli *has been described against penicillins and most of the cephalosporins as well as trimethoprim, sulfamethoxazole, rifampicin, and vancomycin [14–17].

### 2.1. Resistance to Quinolones

The quinolones inhibit the synthesis of bacterial DNA causing cell death. The targets of quinolones are two large bacterial enzymes, DNA gyrase and topoisomerase IV. These enzymes act mutually in bacterial DNA replication, transcription, recombination, and repairing of DNA [18]. There are also other mechanisms of such resistance, including decreased outer membrane permeability and an efflux system [19]. The gyrase and topoisomerase gene products are large enzymatic quaternary structures consisting of two pairs of subunits—GyrA and GyrB (i.e., DNA gyrase), and ParC and ParE (i.e., topoisomerase IV), respectively [20]. Resistance to the fluoroquinolones is mainly due to amino acid(s) substitution(s) in the quinolone resistance-determining region (QRDR) of the corresponding topoisomerase. QRDR is located within the DNA-binding domain on the surface of these enzymes. There are several different single GyrA modifications reported to be associated with fluoroquinolone resistance in *Campylobacter *species: Thr86Ile, Asp90Asn, Thr86Lys, Thr86Ala, Thr86Val, and Asp90Tyr. However, the most frequently observed mutation in quinolone resistant *Campylobacter* is the C257T change in the *gyrA *gene, which leads to the Thr86Ile substitution in the gyrase and confers the high-level resistance to this group of antimicrobials [21]. Other reported resistance-associated mutations include T86 K, A70T, and D90N, which are less common and do not play an important role in quinolone resistance as high as that observed for the Thr86Ile mutation [21, 22].

As it was mentioned above, in *Campylobacter*, fluoroquinolone resistance mechanisms appear to be mainly due to mutations in the *gyrA* gene encoding part of the GyrA subunit of DNA gyrase [23]. It was found that a high-level resistance to ciprofloxacin was conferred by the point mutation Thr86Ile in the *gyrA* gene, which is homologous to Ser83Leu in *Escherichia coli* [24]. Other reported mutations of the *gyrA* region in *C. jejuni* include Thr86Ala which is responsible for a high-level resistance to nalidixic acid and low-level resistance to ciprofloxacin [24, 25]. Double point mutations of the *gyrA* gene together with Asp85Tyr, or Asp90Asn, or Pro104Ser have also been reported [24]. In *C. jejuni *and *C. coli*, the absence of a secondary target for fluoroquinolones infers a situation whereby a unique modification in the GyrA subunit is sufficient to confer a fluoroquinolone-resistant phenotype [21].

The CmeABC multidrug efflux pump has been described as the major efflux mechanism causing antimicrobial resistance to several antimicrobials including the fluoroquinolones and macrolides [26, 27]. CmeABC is coded by an operon consisting of three genes, *cmeA*, *cmeB*, and *cmeC, *which code for a periplasmic fusion protein, an inner membrane drug transporter, and an outer membrane protein, respectively [26]. The CmeABC multidrug efflux pump is the most common efflux system in *Campylobacter* and works in synergy with GyrA mutations in causing fluoroquinolone resistance [28]. Inactivation of the CmeABC efflux pump by insertional inactivation of *cmeB *or with efflux pump inhibitors leads to increased susceptibility to different antibiotics, including those to which *Campylobacter *are intrinsically resistant, showing that CmeABC plays a key role in both intrinsic and acquired resistance of *Campylobacter *[26, 27, 29–31]. Furthermore, it was found that when the efflux pump is blocked, the minimum inhibitory concentration (MIC) values for ciprofloxacin are reduced to the level of susceptible strains even with mutations in the GyrA [28]. 

Quinolone-resistant *Campylobacter *isolates were recognized already during the late 1980s. Then, it was suggested that such resistance was due, at least in part, to acquisition of fluoroquinolone-resistant strains from animal sources [22]. Furthermore, several studies have linked the use of antimicrobials, including fluoroquinolones, as the growth promoters in food animals and therapeutically in the veterinary medicine, with the emergence and spread of resistance among *Campylobacter* strains, with potentially serious influence on food safety as well as veterinary and human health [32–37]. Moreover, the selective pressure of therapeutic fluoroquinolone administration in poultry flocks has been demonstrated to select for ciprofloxacin-resistant campylobacters in poultry that enters the food chain [36, 37]. It was also found that the resistance was not as a result of the spread of a single resistant clone, but that several *Campylobacter* clones were selected by fluoroquinolone treatment [37].

### 2.2. Resistance to Tetracyclines

Resistance to tetracyclines in *Campylobacter *is conferred by the *tet*(*O*) gene, which is widely present in both *C. jejuni* and *C. coli* [38–40]. As it was described in the literature, except the *tet*(*O*) marker no other *tet *resistance genes have been found in *Campylobacter*. Tetracycline binds to Mg^2+^ cations in order to pass through outer membrane porins and then, in the periplasmic space, dissociates from magnesium and moves passively into the cytoplasm to bind to discrete sites on the ribosomal 30S subunit [41]. Its primary antimicrobial effect takes place by direct steric hindrance by binding to the A site in the 30S subunit, thus hindering the movement of transfer RNA and inhibits peptide elongation [42]. The *tet*(*O*) gene, which encodes ribosomal protection proteins (RPPs), is located on a self-transmissible plasmid of a molecular size from 45 to 58 kb [39]. The *tet*(*O*) gene has been shown to confer extremely high-levels of tetracycline resistance (512 mg/L) [43]. Recent study demonstrates that this protein recognizes an open A site on the bacterial ribosome and binds it in such a manner that it induces a conformational change that results in the release of the bound tetracycline molecule [44]. Furthermore, the conformational change persists for an extended period of time, thus allowing for continued protein elongation in an efficient manner [38, 44]. Tetracyclines, which are the subject of RPP mediated resistance, including Tet(O), bind to the ribosome and inhibit accommodation of the aminoacyl tRNA (aa-tRNA) into the ribosomal A site and, therefore, prevent the elongation phase of protein synthesis [45]. Location of the *tet*(*O*) gene on the chromosome has also been reported in 33–76% of tetracycline-resistant *C. jejuni* isolates lacking plasmids in Canada and Australia, respectively [43, 46]. The presence of an insertion element IS607, similar to IS607 found on the chromosome of *Helicobacter pylori*, has been reported on *tet*(*O*)-carrying plasmids [43] and therefore, it is possible that mobile genetic elements other than transmissible plasmids may be involved in the acquisition and dissemination of *tet*(*O*).

Based on the G-C content, sequence homology, codon usage, and hybridization analysis, it seems that the *Campylobacter* *tet*(*O*) gene was probably acquired by horizontal gene transfer from either *Streptomyces*, *Streptococcus, *or *Enterococcus *spp. [40, 47]. The *tet*(*O*) genes showed 75–76% homology sequence with the *tet(M)* gene of *Streptococcus pneumoniae* and have a G to C ratio of 40% [39].

### 2.3. Resistance to Macrolides

Macrolides are mostly produced by *Streptomyces *and related bacteria. Erythromycin is a natural product of *Saccharopolyspora erythraea* and it is the first macrolide antimicrobial isolated. The macrolides are widely used antimicrobial agents and considered to be safe and effective drugs. Their antimicrobial spectrum covers most of Gram-positive and the Gram-negative microorganisms, including *Campylobacter*.

Macrolides interrupt protein synthesis in bacterial ribosome by targeting the 50S subunit and inhibit bacterial RNA-dependent protein synthesis [48, 49]. Structural studies demonstrated that the 23S rRNA nucleotides 2058 and 2059 act as key contact sites for macrolide binding. The binding of the macrolide antimicrobial leads to conformational changes in the ribosome and subsequent termination of the elongation of the peptide chain [50]. The chromosome of *Campylobacter *contains three copies of the 23S rRNA gene [51]. In erythromycin-resistant strains, generally all copies carry macrolide resistance-associated mutations, but the co-existence of wild-type alleles does not seem to affect the resistance level [43, 52].

Macrolide resistance in *Campylobacter *is the result of modification of the ribosome target binding site by mutation of the 23S rRNA or changes in resulting proteins at the site rather than target methylation or enzymatic drug modification seen in other bacterial species [47, 53]. Base substitutions at positions 2074 and 2075 of the adenine residues in all three copies of the 23S rRNA gene (*rrnB *operon) in *Campylobacter *are the most common mutations conveying erythromycin resistance [54]. The A2074C, A2074G, and A2075G mutations are found to confer a high-level resistance to macrolide antibiotics (erythromycin MIC > 128 mg/L) in *C. jejuni *and *C. coli*. Resistance to erythromycin tends to correspond with cross-resistance to other macrolides (e.g., azithromycin and clarithromycin) as well as related drugs of the lincosamide (e.g., clindamycin) and streptogramin groups [55].

Resistance to macrolides among *Campylobacter *isolates may also be caused by modifications of the ribosomal proteins L4 and L22. Several modifications have been reported and it is possible that they might be associated with low-level resistance to the macrolides. However, the exact role of these L4 and L22 modifications (mutations, insertions, and deletions) is still not clear [20, 56–58]. 

Efflux is another common mechanism causing macrolide resistance in *Campylobacter* bacteria where at least eight different efflux systems have been identified. One of them is CmeABC multidrug efflux pump that works in synergy with specific mutations, even in the absence of any other factor affecting resistance [20, 56]. There is data suggesting that interplay between efflux activity and mutations in the 23S rRNA gene contributes to high-level macrolide resistance in some *Campylobacter *isolates [58]. It was found that even in the highly resistant *Campylobacter *strains with the A2074G or A2075G mutation, inactivation of CmeABC also significantly reduced the resistance level to macrolides, suggesting that this efflux system functions synergistically with target mutations [56, 59–61]. In isolates with low level of erythromycin resistance (MICs 8–16 mg/L), no mutations have been detected in the target gene [20], and in these isolates the inactivation of CmeABC leads to restored susceptibility to erythromycin, suggesting the involvement of CmeABC in the intrinsic resistance of *Campylobacter *[59, 61]. In strains with a high erythromycin resistance level (MIC > 128 mg/L), the resistance is associated with a mutation in the *23S rRNA* gene [20]. In these isolates, the inactivation of CmeABC leads to 2- to 4-fold decrease in erythromycin resistance, implying synergistic action with the target mutations in achieving acquired macrolide resistance [59, 61, 62]. Additionally, the synergy between the CmeABC efflux pump and mutations in the ribosomal proteins L4 (G74D) and L22 (insertions at position 86 or 98) was also shown to confer macrolide resistance in *C. jejuni *and *C. coli *[56, 57].

### 2.4. Resistance to Aminoglycosides

Aminoglycoside resistance genes are present in many bacterial species and commonly encode proteins that modify these antimicrobials. Aminoglycosides (e.g., gentamycin, streptomycin, and kanamycin) act by binding to the decoding region in the A-site of the bacterial ribosomal 30S subunit. This interaction results in aberrant proteins by interfering with accurate codon-anticodon recognition and in disruption of elongation of the proteins by inhibiting the translocation of tRNA from the A-site to the P-site [63]. Multiple aminoglycoside modifying enzymes, including aminoglycoside phosphotransferase types I, III, IV, and VII, aminoglycoside adenyltransferase, and 6-aminoglycoside adenyltransferase, have been described in *Campylobacter *[64]. Aminoglycoside resistance is mediated by enzymatic modification that diminishes affinity of aminoglycosides for the rRNA A-site [65]. These enzymes fall into three classes: aminoglycoside acetyltransferases, aminoglycoside adenyltransferases, and aminoglycoside phosphotransferases, each of which has its own characteristic modification sites and substrates [66]. However, all three enzymes act via a similar mechanism: the production of a 30-O-aminoglycoside phosphotransferase [23]. This protein is the most common enzyme found in *C. jejuni *and *C. coli *[67, 68].

A kanamycin-resistance phosphotransferase gene, apha-7, was also identified on a 14-kb *C. jejuni* plasmid [69]. The DNA sequence of these genes demonstrated 55% identity with the apha-3 gene from streptococci; however, it showed a 32.8% G/C ratio suggesting that the apha-7 gene may be unique in the *Campylobacter* genus [69]. Kanamycin resistance is often mediated by a plasmid that also encodes tetracycline resistance [39] and has been reported to be transferred along with tetracycline resistance by conjugation between *Campylobacter* strains [43].

### 2.5. Resistance to Other Antimicrobial Agents

Mechanisms of *Campylobacter* resistance to some betalactams such as ampicillin and some of the expanded-spectrum cephalosporins are variable and not very clearly defined [70–72]. Generally, with the exception of some carbapenems, the majority of *Campylobacter* strains are considered to be resistant to betalactam antimicrobial agents, especially the penicillins and narrow-spectrum cephalosporins. Betalactam antimicrobials bind to penicillin binding proteins and disrupt peptidoglycan crosslinking during bacterial cell wall formation and which leads to cell death [73]. Furthermore, alterations in the membrane structure or in porin proteins and the efflux pump system can cause resistance to this antimicrobial group [73–78]. While *Campylobacter* are generally inherently resistant to many betalactams, they remain susceptible to, for example, amoxicillin and ampicillin [72]. A vast majority of *C. jejuni *and *C. coli *isolates are able to produce betalactamases, which inactivate the betalactam molecule by hydrolysing the structural lactam ring [72, 78].

Another mechanism for betalactam resistance in *Campylobacter *is the action of efflux pumps. Several studies have demonstrated a significant increase in susceptibility to ampicillin in CmeABC-inactivated *C. jejuni *mutants and a decrease in susceptibility in CmeABC-overexpressing mutants [26, 27, 30], but this phenomenon was less pronounced in ampicillin-resistant and betalactamase-positive strains [26].

Chloramphenicol inhibits bacterial protein biosynthesis by preventing peptide chain elongation. It binds reversibly to the peptidyl transferase centre at the 50S ribosomal subunit [79]. Chloramphenicol resistance is conferred by a plasmid carried *cat *gene that encodes acetyltransferase, which modifies chloramphenicol in a way that prevents it from binding to ribosomes [80]. Although chloramphenicol resistance in *Campylobacter *is rare, a plasmid-carried chloramphenicol resistance gene has been reported in *C. coli *[81].

Sulphonamide resistance in *C. jejuni* is also chromosomally mediated through mutational substitution of four amino acid residues in the enzyme dihydropteroate synthetase (DHPS), resulting in a reduced affinity for sulphonamides. Sulphonamides compete with PABA (4-aminobenzoic acid**)** for DHPS, thereby preventing PABA from being incorporated into folic acid [23].

## 3. Factors Influencing Antimicrobial Resistance of *Campylobacter*

Since campylobacteriosis is a zoonotic foodborne disease, the presence of resistant strains in the food chain also has an influence on human infections. One of the main factors influencing antimicrobial resistance, especially to fluoroquinolones and macrolides, is the use of these antimicrobial agents in animal production. In the early 1990s, when enrofloxacin was introduced into animal production in Asia and in Europe, at the same time fluoroquinolone resistance started to increase among human *Campylobacter* isolates [32]. The same phenomenon was observed in UK and USA after the approval of the use of fluoroquinolones in veterinary medicine [13, 82]. In many countries, where fluoroquinolone use in animal production is low, the incidence of fluoroquinolone-resistant strains has remained moderate or low. For example in Australia, where application of fluoroquinolones in animal production is prohibited, *Campylobacter *strains isolated from pigs are mainly ciprofloxacin-susceptible [83]. The same findings have been described in Finland and Sweden [84, 85]. Furthermore, in Denmark, the use of fluoroquinolones in animal husbandry has been restricted since 2003 and the recent study reported a significantly higher resistance to ciprofloxacin, nalidixic acid, and tetracycline in *C. jejuni *from imported poultry meat compared to Danish poultry meat [86].

In case of macrolides, the use of these antimicrobials in animal production as therapeutic or growth-promoting agents has been considered to be one important factor in the selection of erythromycin-resistant *Campylobacter *strains. However, acquisition of erythromycin resistance in *Campylobacter *is a stepwise process and requires prolonged exposure in contrast to the rapidly envolving fluoroquinolone resistance. Schönberg-Norio et al. [84] studied the effect of tylosin given to poultry at subtherapeutic and therapeutic concentrations and observed that after the drug administration, the overall erythromycin resistance rate among *C. coli *isolates was at a higher frequency than among *C. jejuni *strains. Furthermore, the erythromycin resistance rate was higher when tylosin was given at subtherapeutic than at therapeutic concentrations. Similar observations have been described by Juntunen et al. [87] who studied the effects of tylosin treatment of pigs and observed that it selected high-level resistance to erythromycin, as well as resistance to ciprofloxacin and nalidixic acid. Lin et al. [59] studied the frequency of spontaneous mutations to an erythromycin resistant phenotype and found that both *C. jejuni *and *C. coli *have extremely low rates of spontaneous mutations under *in vitro *culture conditions.

## 4. Epidemiology of Fluoroquinolone and Macrolide Resistance in *Campylobacter*

Resistance of *Campylobacter* to fluoroquinolones was first reported in the late 1980s and since then, it has been increasing in many countries [2]. As it was mentioned above, the resistance appeared at the same time as the introduction of these agents in animal production and veterinary medicine [23]. Since then, the fluoroquinolone resistance among *Campylobacter* isolates of human, animal, and food of animal origin is common. For example, in Asia countries (Thailand and India), 80% and 77% of *Campylobacter *isolates, respectively, have been reported to be resistant to fluoroquinolones [88, 89]. Even higher resistance rates to ciprofloxacin have been reported in China for *C. coli *strains isolated from swine (95.8–99% of the isolates) [90]. Similar incidence of resistance has also been observed in the United Arab Emirates (85.4%) [91] and South Africa (91%) [92]. In Europe, the emergence of fluoroquinolone resistance evaluated between 1993 and 2003 in Spain showed a statistically significant increase for nalidixic acid (46.7% of the isolates) and ciprofloxacin (52.2%) [93]. Similarly, in the United Kingdom, resistance to fluoroquinolones in *Campylobacter* isolates was observed after the approval of the use of the antimicrobials as growth promoters in food producing animals [82]. An increasing resistance to fluoroquinolones among *Campylobacter* strains isolated from poultry was also observed in Poland where during 1994–1996 and 2005–2008 47.9% and 90.2% of such isolates were resistant to ciprofloxacin, respectively [94]. Furthermore, in 2001 in Germany, the proportion of human *Campylobacter* isolates resistant to ciprofloxacin was 41–46%, while 42% and 71% of chicken strains of *C. jejuni* and *C. coli*, respectively, were resistant to ciprofloxacin [95]. In the United States, the introduction of sarafloxacin and enrofloxacin in the mid-1990s for use as growth promoters in poultry flocks also contributed to fluoroquinolone resistance, with resistance among *Campylobacter* isolates from humans increasing from 1.3% in 1992 to 10.2% in 1998 [13]. On the other hand, there are also studies showing a low (Grenada; 9.4% of the strains) or even lack (Norway, Finland) of fluoroquinolone-resistant *Campylobacter *isolates [96–98]. A study from Denmark demonstrated that resistance rates to ciprofloxacin, nalidixic acid, and tetracycline were significantly higher in travel-associated infections compared to infections acquired domestically, and that the occurrence of resistance increased during the years 2006 and 2007 [86]. Similar observations were made for Finland where after 1990 the rate of ciprofloxacin resistance has clearly increased, and between 1998 and 2000, the majority of strains isolated from Finnish patients after travelling to Spain or Thailand (70% and 79%, resp.) were resistant to ciprofloxacin [99]. Furthermore, quinolone resistance among strains of *Campylobacter* in Australia remains low and this is attributed to the infrequent use of antibacterials for the treatment of diarrhoea and the regulatory prohibition on the use of fluoroquinolones in food-producing animals [100, 101]. It has been shown that induction of fluoroquinolone resistance during treatment was also well recognized and had been reported [102, 103]. A predicted 10% of patients treated with a fluoroquinolone for *Campylobacter* enteritis reportedly harbour quinolone resistant strains [32] and development of resistance has been reported within 24 h of treatment with fluoroquinolones, but prolonged therapy, especially in the immunocompromised, is also a risk factor [22].

The macrolides are now generally considered to be the optimal drug for treatment of *Campylobacter* infections; however, resistance to macrolides in human isolates in some countries is becoming a major public health concern. The macrolide resistance among *Campylobacter* strains has remained at a low and stable level for a long time. However, there is also evidence from some parts of the world that resistance rates to erythromycin, and other macrolides in these bacteria are slowly increasing [104, 105]. Since, as mentioned above, fluoroquinolone resistance is common, the macrolides have become important in the treatment of campylobacteriosis, which also has influence on the development of resistance. In a study performed in China, *Campylobacter* isolates recovered from poultry showed the resistance rates to erythromycin, azithromycin and clindamycin of 8.9%, 26.7%, and 13.9%, respectively, for *C. jejuni *isolates and even more for *C. coli* strains, that is, 100%, 98.1%, and 100%, respectively [106]. In another study from the same country, high resistance rates to macrolides were also reported for *C. coli *strains isolated from swine (37.9–54.7% of the strains were resistant to erythromycin) [90]. In Poland, a statistically significant increase in the percentage of *Campylobacter* strains medium resistant to erythromycin, which had been isolated from poultry between years 1994–1996 and 2005–2008 was observed (49.3% and 88.9%, resp.) [107]. On the other hand, several countries still report a low level of erythromycin resistance among *Campylobacter* isolates from human clinical samples.

## 5. Development and Transmission of Antibiotic Resistance in *Campylobacter*

Mutations play a major role in development of *Campylobacter *resistance. Several mechanisms have been reported to contribute to the emergence of these mutations. It was demonstrated that *C. jejuni *lacks many of the genes encoding DNA repair molecules present in other bacteria, for example, *mutH *and *mutL *(methyl-directed mismatch repair), *sbcB *(recombination repair), *phr *(repair of pyrimidine dimers), and *vsr *(very short patch repair), as well as genes protecting from UV-induced mutagenesis (*umuCD*) and alkylating agents (*ada *gene), facilitating the appearance of mutations [51, 108, 109].

Besides spontaneous mutations, *Campylobacter *are also able to acquire resistance determinants by natural transformation, transduction, or conjugation, for example, conjugation of *tet*(*O*)-carrying plasmids [64]. In the presence of antimicrobial selection pressure, the bacteria containing these resistance determinants overgrow the susceptible bacteria. It was estimated that 28% of human patients treated with a fluoroquinolone will develop resistance against these antimicrobials [66, 110]. In addition, the emergence of *Campylobacter *resistance in human clinical samples has been shown to be closely connected to antimicrobial resistance found in animals [111]. The transmission of resistant *Campylobacter *strains has been analyzed in several studies, where an association between resistant animal and human strains has been investigated [32]. Antimicrobial agents with clinical significance to treating campylobacteriosis in humans, such as macrolides, fluoroquinolones, and tetracyclines, have all been used extensively in farm animals as therapeutic agents, prophylactics, or growth promoters. Since the beginning of large-scale use of fluoroquinolones in the early 1990s, the number of resistant *Campylobacter *strains has clearly increased in both farm animals and humans. In addition to the mutation-based mechanisms, *Campylobacter *can also acquire antibiotic resistance determinants via horizontal gene transfer (HGT). Transfer of DNA between *Campylobacter *strains has been shown both *in vitro* in bacterial cultures [54, 112] and *in vivo* in chicken intestine [55, 113]. HGT is mediated by natural transformation, conjugation, and transduction, all of which can be found in *Campylobacter*. Conjugation plays a major role in the transfer of plasmid-mediated resistance, such as the *tet*(*O*) gene, while natural transformation may be a major mechanism for the transfer of chromosomally encoded resistance (e.g., fluoroquinolone and macrolide resistance). Multiple plasmids have been reported in *Campylobacter*, some of which can be transmitted by conjugation [46, 114–116]. Many of the conjugative plasmids carry genes mediating resistance to tetracyclines [46, 117] and aminoglycosides [116, 118]. It was reported that the transfer of a conjugative plasmid carrying the *tet*(*O*) gene occurred between *C. jejuni *strains in the intestinal tract of chickens [55]. Considering the high prevalence of conjugative *tet*(*O*) plasmids, it is possible that conjugation has contributed to the spread of tetracycline resistance in *Campylobacter*.

Integrons and mobile genetic elements, such as transposons and insertional sequences, are important players for the transmission and spread of antibiotic resistance genes in bacteria [119, 120]. However, these elements are not common in *Campylobacter *and do not appear to play a major role in the horizontal transfer of antibiotic resistance in *Campylobacter*. Class I integrons, which are the most common integrons associated with antibiotic resistance, were reported in both *C. jejuni *and *C. coli *and were found to carry aminoglycoside resistance genes (*aadA2 *and *aacA4*) [64, 77, 117, 120, 121].

Resistance to fluoroquinolones and macrolides in *Campylobacter *occurs spontaneously due to mutations in target genes. It was shown *in vitro* that the frequencies of emergence of fluoroquinolone-resistant mutants range from approximately 10^−6^ to 10^−8^/cell/generation [122]. Different point mutations occur in the QRDR region of the *gyrA *gene and confer varied levels of resistance to fluoroquinolones [122]. Thus, the measured frequencies of emergence of fluoroquinolone resistance vary with the concentration of antibiotics used in the media for mutant enumeration. As it was mentioned above, the higher expression of the *cmeABC *gene increases the frequency of emergence of resistant mutants. In addition, Mfd (Mutant Frequency Decline), a transcription repair coupling factor involved in strand-specific DNA repair, promotes the emergence of fluoroquinolone resistant mutants in *Campylobacter *[123]. On the other hand, inactivation of the *mfd *gene resulted in a 100-fold reduction in the number of spontaneous mutants resistant to ciprofloxacin, while overexpression of *mfd *increased the mutant numbers. Several studies have demonstrated the rapid development of fluoroquinolone resistant mutants in chickens originally infected with fluoroquinolone-susceptible *C. jejuni* but treated with enrofloxacin [28, 34–36, 124]. Fluoroquinolone-resistant *Campylobacter *mutants were detected in feces already after 24 h of the treatment, and the resistant bacterial population colonized the intestinal tract of the birds. Thus, treatment of *Campylobacter*-infected birds does not eradicate the organisms but converts an originally fluoroquinolone-susceptible population to fluoroquinolone-resistant *Campylobacter*. Since contaminated poultry meat is a main source of human *Campylobacter *infections, the fluoroquinolone resistant *Campylobacter *developed in poultry can be transmitted to humans via the food chain. The development of fluoroquinolone-resistant *Campylobacter *from antibiotic treatment was also observed in pigs infected with *C. coli *and human patients infected with *C. jejuni *[103, 125, 126]. Thus, these observations indicate that *Campylobacter *is highly adaptable to fluoroquinolone treatment.

In contrast to fluoroquinolone resistance, the mutation frequency for macrolide resistance in *Campylobacter *is low (~10^−10^/cell/generation) and is approximately 10,000-fold lower than that of fluoroquinolone resistance [59, 122]. The mutants obtained by single-step selection tend to have low to intermediate levels (MIC 8–64 mg/L) of resistance to erythromycin and usually harbor mutations in the L4 and L22 proteins [57, 59, 119]. On the other hand, the mutations in 23S rRNA seem to require stepwise selection, that is, increased antibiotic concentrations and/or prolonged exposure to macrolide antibiotics [57, 59]. Once acquired, most 23S rRNA mutations confer a high-level of resistance to erythromycin (MIC ≥ 512 mg/L) and can be stably maintained in the absence of macrolides [22, 57, 58, 127–129].

Another unique feature of macrolide resistance in *Campylobacter *is the slow development of resistant mutants under antibiotic treatment. Using *Campylobacter*-infected chickens it was shown that therapeutic treatment of *Campylobacter*-infected birds with tylosin in drinking water did not select for erythromycin-resistant *Campylobacter*, even after several antibiotics administration [59]. This observation is in contrast to the development of fluoroquinolone resistance, which occurs rapidly in birds treated with enrofloxacin. However, when the same antimicrobial (tylosin) was given to *Campylobacter*-infected birds daily as a feed additive, after several weeks of exposure, erythromycin-resistant *Campylobacter *emerged in the chickens [59].

## 6. Conclusions and Future Perspectives

*Campylobacter *infections typically cause self-limiting gastroenteritis and the most important treatment is to avoid dehydration. Antimicrobial treatment is needed only in the most severe and persisting infections or infections of young children, pregnant women as well as old and immunocompromised patients [2, 9, 10, 130, 131]. The incidence of *Campylobacter* infection in humans is increasing in the European Union and other parts of the world. Trends in antimicrobial resistance have shown a clear correlation between use of antibiotics in the veterinary medicine and animal production and resistant isolates of *Campylobacter* in humans. Globally, the incidences of resistance to several important antibiotics useful in the treatment of campylobacteriosis are increasing and multiple resistance patterns to several classes of antibiotics are emerging. In many countries, resistance in *Campylobacter* to the fluoroquinolones has limited its usefulness as a drug of choice in the treatment of human infection, although in some countries such as Australia, the fluoroquinolones remain an effective antibiotic. Similarly, resistance to macrolides (erythromycin) is increasing for several *Campylobacter* isolates, particularly in *C. coli*; however, the incidence of erythromycin resistance in human strains is still relatively low and thus erythromycin should be regarded as the drug of choice in the treatment of campylobacteriosis. Furthermore, gentamicin also remains effective against campylobacters, although it would normally be considered only for serious *Campylobacter* infections. Macrolides are still the most effective antibiotics against *Campylobacter *infections, but the rising trend of erythromycin resistance in *C. coli *and *C. jejuni *in some regions requires prudent use of this class of antibiotics. Additional studies are needed to understand how macrolide resistant *Campylobacter *emerge under selective pressure. Since several antimicrobials are no longer effective in the clinical treatment of campylobacteriosis, new generation of antibiotics and novel treatment schemes, which avoid the selection of fluoroquinolone-resistant mutants, should be evaluated. Modern molecular approaches, such as genomics and proteomics, are expected to provide new insights into the molecular mechanisms involved in the development of antimicrobial resistance in *Campylobacter*. 
